# A Case of Miliary Tuberculosis Complicated by Knee Joint Tuberculosis

**DOI:** 10.1155/crdi/9328618

**Published:** 2026-04-24

**Authors:** Saiki Yoshimura, Kentaro Satomi, Kohei Fujita, Hiromasa Tachibana, Yuta Okada, Naoki Fujimoto, Shogo Toyama, Takanori Ito, Takuma Imakita, Issei Oi, Osamu Kanai, Kiminobu Tanizawa

**Affiliations:** ^1^ Division of Respiratory Medicine, Center for Respiratory Diseases, National Hospital Organization Kyoto Medical Center, Kyoto, Japan, hosp.go.jp; ^2^ Department of Orthopaedic Surgery, National Hospital Organization Kyoto Medical Center, Kyoto, Japan, hosp.go.jp; ^3^ Department of Respiratory Medicine, National Hospital Organization Minami-Kyoto Hospital, Joyo, Japan

**Keywords:** debridement, knee joint TB, miliary TB, orthopaedic TB

## Abstract

This case report details a 75‐year‐old male diagnosed with miliary tuberculosis (TB) who subsequently developed tuberculous arthritis in his left knee. The patient was initially admitted for a hemorrhagic gastric ulcer, where chest imaging and positive cultures from multiple sites confirmed miliary TB. Standard antitubercular therapy was started, but 6 weeks later, he presented with worsening pain and swelling in his left knee. A joint aspiration and MRI confirmed tuberculous arthritis with an abscess, which necessitated surgical debridement in addition to the drug regimen. We emphasize that orthopaedic TB often requires surgical intervention alongside medication for successful treatment. While total knee arthroplasty can be an effective treatment for joint destruction, the previous report suggests that it should be delayed until after a prolonged course of antitubercular therapy (e.g., 12 months or more) to prevent recurrence.

## 1. Introduction

Miliary tuberculosis (TB) is a severe, disseminated form of TB, with a global epidemiology that has seen a resurgence, particularly in developing nations and among immunocompromised populations, such as those with HIV/AIDS [1]. The infection spreads hematogenously, affecting multiple organs. Treatment is a prolonged regimen of multiple antitubercular drugs. The total duration of treatment is often longer than for pulmonary TB, sometimes extending to 12 months or more depending on the patient’s response and the sites of involvement [2]. Mortality rates can be high without timely and appropriate treatment, ranging from 15% to 30%, even with therapy [2]. Factors affecting prognosis include the patient’s age, nutritional status, immune status, and the presence of complications like acute respiratory distress syndrome or multidrug resistance [2]. Early diagnosis and prompt initiation of a correct drug regimen are crucial for a favourable outcome. Musculoskeletal TB accounts for approximately 10% of extrapulmonary TB cases. Tuberculous spondylitis is the most common type of skeletal TB, accounting for about half of musculoskeletal TB cases, while the proportion of knee joint TB is not particularly high at 8% [3]. The WHO recommends a six‐month standard treatment regimen for new TB patients. It also says the recommendation applies to extrapulmonary TB, except TB of the central nervous system, bone or joint, for which some expert groups suggest longer therapy [2]. There is no consensus on the optimal treatment duration for joint TB. Surgical debridement or synovectomy is often required in conjunction with antituberculosis therapy to remove diseased tissue and improve joint function [4, 5]. Here, we experienced a case of miliary TB complicated by knee joint TB.

## 2. Case Presentation

A 75‐year‐old male was urgently admitted to the gastroenterology department of our hospital owing to a hemorrhagic gastric ulcer. Chest x‐ray and computed tomography (CT) scan at admission (Figures 1(a) and 1(b)) revealed multiple granular shadows in the bilateral lung fields, raising suspicion of pulmonary TB and tuberculous pleurisy. Following emergency endoscopic haemostasis for a hemorrhagic gastric ulcer, a consultation was requested from the respiratory medicine department regarding pulmonary lesions.

FIGURE 1(a) Chest x‐ray revealed multiple nodular shadows in bilateral lung fields. (b) A chest CT scan revealed multiple nodular shadows in bilateral lung lobes and bilateral pleural effusions, suggesting miliary tuberculosis and tuberculous pleurisy. (c and d) A knee MRI was performed, revealing fluid accumulation within the knee joint and surrounding tissues. Multiple nodules with low signal intensity on T1‐ and T2‐weighted images were observed within this fluid accumulation. (e and f) The surface of the left knee joint showed redness and swelling (e) and debridement and irrigation were performed during surgery (f).

**Figure:**
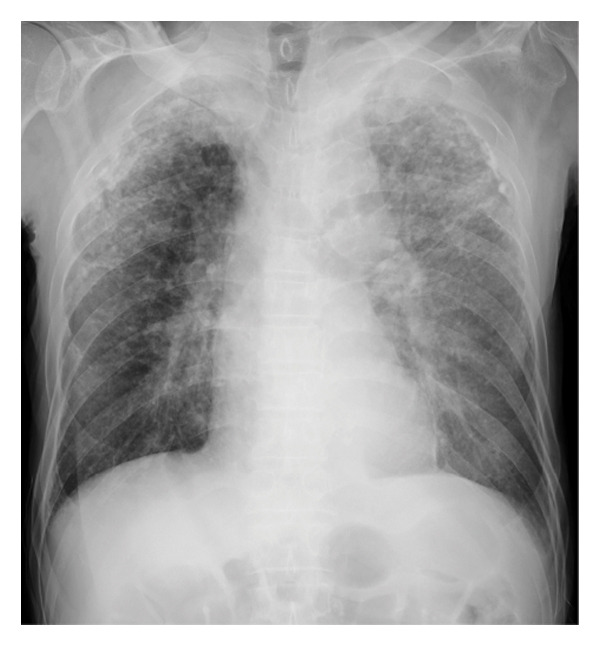
(a)

**Figure:**
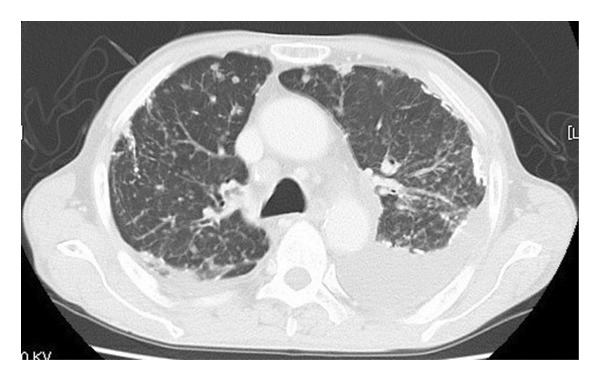
(b)

**Figure:**
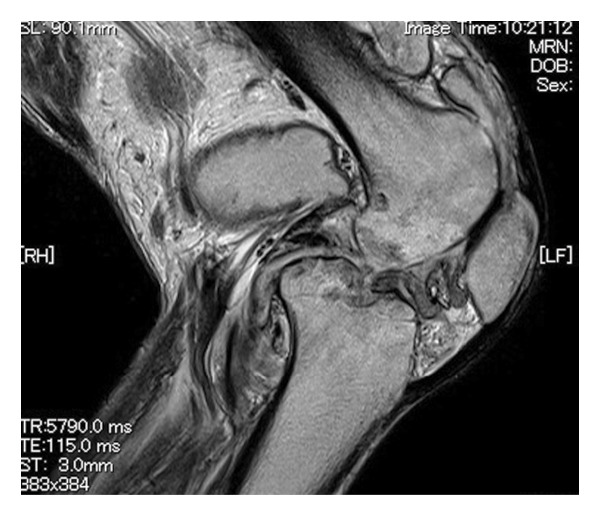
(c)

**Figure:**
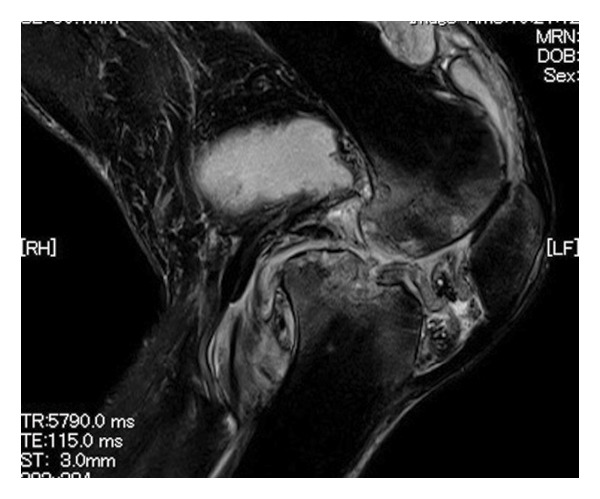
(d)

**Figure:**
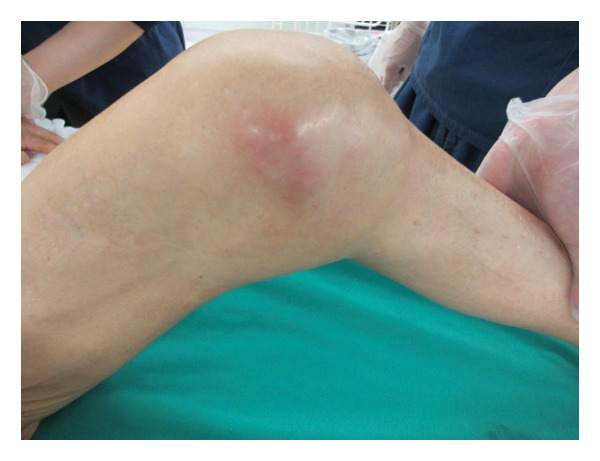
(e)

**Figure:**
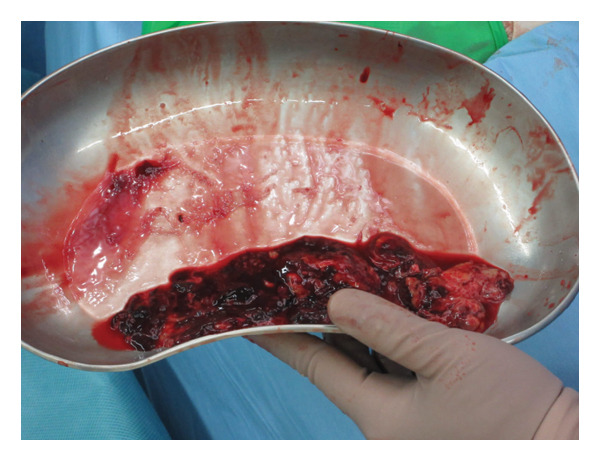
(f)

He had no significant past medical history though his maternal uncle and aunt had a history of treatment for active pulmonary TB. He is a current smoker with 50 pack‐year. Prior to admission, he had no difficulty with activities of daily living. His sputum cultures, pleural fluid cultures, blood cultures, urine cultures, and gastric fluid cultures revealed positive results of acid‐fast bacilli (AFB) smears and PCR confirmed *Mycobacterium tuberculosis*, leading to a diagnosis of pulmonary TB, tuberculous pleurisy and miliary TB. We initiated standard treatment of combined isoniazid, rifampicin, ethambutol and pyrazinamide. AFB smears became negative after 3 weeks. Drug susceptibility test showed good susceptibility to all standard treatment drugs after 5 weeks.

At the 6th week, pain, swelling and heat in the left knee worsened, so we consulted orthopaedics in the 7th week. A joint aspiration of the left knee was performed 6 weeks after the start of treatment and showed positive AFB smear and TB‐PCR, leading to a diagnosis of tuberculous arthritis. We confirmed this TB exhibited good drug susceptibility to isoniazid, rifampicin, ethambutol and pyrazinamide. At the 9th week, worsening swelling and heat in the knee joint was noted. A left knee magnetic resonance imaging (MRI) revealed fluid accumulation within the knee joint and surrounding tissues. Multiple nodules with low signal intensity on T1‐ and T2‐weighted MRI images were observed within this fluid accumulation (Figures 1(c) and 1(d)). An abscess of knee joint due to TB was strongly suspected. Treatment plans were discussed in the orthopaedic department, and debridement of the lesion was performed within a few days after the discussion. Incisions of the skin were made at five sites, debridement and flushing of the lesion were performed and drains were placed (Figures 1(e) and 1(f)). Figure 2(a) shows macroscopic findings of tissue excised from the left knee joint. Histopathology revealed extensive necrotic tissue on a background of haematoxylin and eosin staining, with scattered multinucleated giant cells and epithelioid cells, surrounded by inflammatory cells (Figure 2(b)). Ziehl–Neelsen staining revealed scattered AFB, consistent with the pathological findings of tuberculous arthritis (Figure 2(c)). The drain was removed on the third postoperative day. After surgery, the fever subsided, and CRP gradually decreased. Knee symptoms improved, allowing the patient to get out of bed. The patient transitioned to maintenance therapy at the 10th week. He continued rehabilitation and was discharged home approximately 9 months after treatment initiation. Total knee arthroplasty (TKA) treatment is scheduled to be performed after 12 months of TB treatment.

FIGURE 2(a) Macroscopic findings of tissue excised from the left knee joint. (b) Histopathology revealed extensive necrotic tissue on a background of haematoxylin and eosin staining, with scattered multinucleated giant cells and epithelioid cells, surrounded by inflammatory cells. (c): Ziehl–Neelsen staining revealed scattered acid‐fast bacilli, consistent with the pathological findings of tuberculous arthritis.

**Figure:**
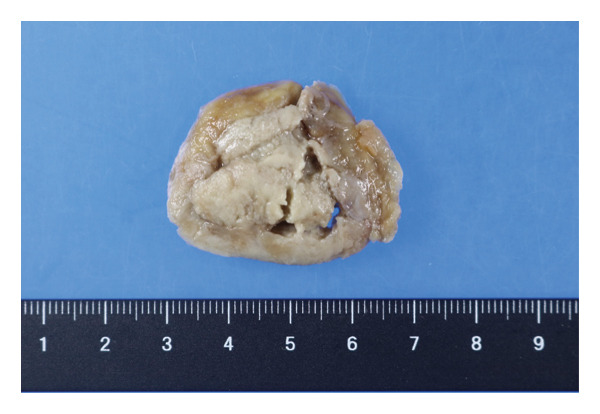
(a)

**Figure:**
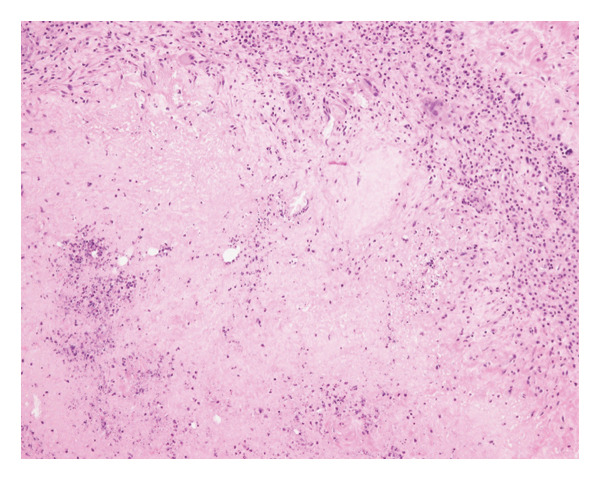
(b)

**Figure:**
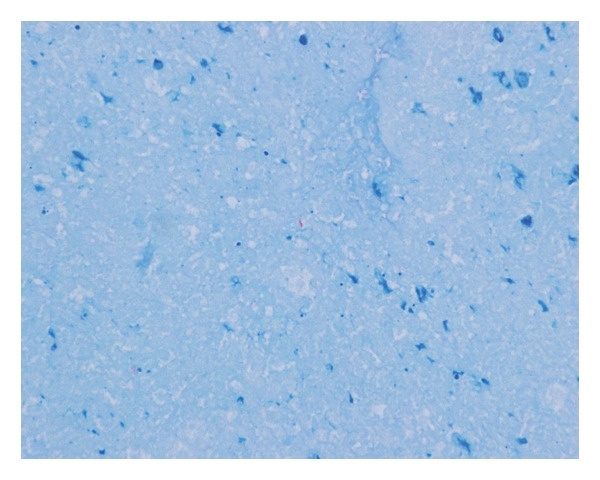
(c)

## 3. Discussion

Typically, individuals who develop miliary TB are often in some state of immunosuppression [5]. We screened for the presence of severe diabetes, HIV infection, and malignant disease after admission, but none of these conditions were present. However, this patient was initially admitted for a haemorrhagic gastric ulcer and presented with cachexia. We, therefore, consider that the state of malnutrition resulting from this severe gastric ulcer was a major contributing factor to the development of miliary TB. In cases of TB of the knee where joint swelling is significant due to abscess formation, early aspiration and drainage are desirable to prevent skin necrosis and fistula formation. Surgical treatment often involves synovectomy and curettage of the lesion, with some reports also describing two‐stage TKA. TKA for TB of the knee has been reported to yield favourable outcomes [6]. One contributing factor is that, unlike common bacteria, *M. tuberculosis* rarely colonizes metal surfaces and has difficulty forming biofilms [7]. Conversely, there have been reports of TB recurrence, and there is no consensus on the optimal timing for performing TKA for TB knees [6]. According to Su et al., half of knee joint TB cases recurred during TKA, suggesting that preoperative TB treatment for at least several months is important [8]. Furthermore, postoperative TB treatment is also considered important for preventing the recurrence of TB. Most reports recommend a duration of 12–18 months [6]. At present, there is no comment actively recommending an 18‐month TB treatment period [9]. On the other hand, extending the standard treatment period by 6 months is recommended for extrapulmonary TB and miliary TB. While there is no standardised treatment period for joint TB in Japanese guideline [9], which is the focus here, considering that the primary site of the disease is often within the joint cavity where drugs are difficult to deliver, an 18‐month treatment period has been mentioned by the several reports [6, 8]. Although the guideline of the European Bone and Joint Infection Society do not recommend 18 months of treatment [10], and the duration of treatment should be carefully considered on a case‐by‐case basis, following multidisciplinary discussions, we determined that 18 months of treatment was appropriate for our case at this time. Consequently, we plan to extend TB treatment following TKA.

## 4. Conclusion

In conclusion, since orthopaedic TB often proves difficult to treat with anti‐TB therapy alone, it is essential to always consider adding surgical treatment. Early consultation with an orthopaedic surgeon is very important. TKA may be considered in some cases, but based on previous reports, it is recommended to perform it during the maintenance phase or later to prevent recurrence.

## Author Contributions

All the authors contributed to the manuscript. The first draft of the manuscript was written by Saiki Yoshimura, and Kohei Fujita revised the manuscript.

## Funding

No funding was obtained for this study.

## Disclosure

All the authors have read and approved the final manuscript.

## Consent

The authors declare that written informed consent was obtained for the publication of this manuscript and accompanying images using the consent form provided by the Journal.

## Conflicts of Interest

The authors declare no conflicts of interest.

## Data Availability

The data that support the findings of this case report are available from the corresponding author upon reasonable request.
